# Can texture analysis of T2-weighted MRI be used to predict extrathyroidal extension in papillary thyroid carcinoma?

**DOI:** 10.1097/MD.0000000000035800

**Published:** 2023-11-03

**Authors:** Chengjia Qian, Shan Chen, Li Liu, Weiqiang Dou, Shudong Hu, Heng Zhang

**Affiliations:** a Department of Gastrointestinal Surgery, Affiliated Hospital of Jiangnan University, Wuxi, China; b Wuxi School of Medicine, Jiangnan University, Wuxi, China; c Big Data Center, Affiliated Hospital of Jiangnan University, Wuxi, China; d GE Healthcare, MR Research China, Beijing, China; e Department of Radiology, Affiliated Hospital of Jiangnan University, Wuxi, China.

**Keywords:** magnetic resonance imaging, neoplasm staging, papillary thyroid carcinoma, texture analysis, thyroid cancer

## Abstract

Determining the presence of extrathyroidal extension (ETE) is important for established of different surgical protocol and postoperative patient management in patients with papillary thyroid carcinoma (PTC). The correlation relationship between texture features from T2-weighted imaging (T2WI) and ETE has not been explored extensively. This study aimed to explore the value of T2-weighted magnetic resonance imaging – based whole tumor texture analysis in predict extrathyroidal extension with PTC. In this retrospectively study, 76 patients with pathologically proven PTC were recruited, who received surgical resection and underwent preoperative thyroid magnetic resonance imaging. Based on histo-pathologically findings, patients were classified into ETE and no ETE groups. ETE group was further divided into 2 subgroups (minimal ETE and extensive ETE). Whole-tumor texture analysis was independently performed by 2 radiologists on axial T2WI images. Nine histogram and gray-level co-occurrence matrix (GLCM) texture features were automatically extracted. Univariate and multivariate analysis were performed to determine risk factors associated with ETE. Predictive performance was evaluated by receiver operating characteristic (ROC) analysis. Interobserver agreement, confirmed by intraclass correlation coefficients (ICCs) ranging from 0.78 to 0.89, was excellent for texture analysis between 2 radiologists. T2WI image derived entropy, standard deviation, energy and correlation have significant difference between PTC with and without ETE (all *P* < .05). Among these, entropy showed the best diagnostic efficiency with the area under ROC curve of 0.837, diagnostic threshold of 5.86, diagnostic sensitivity and specificity of 81.5% and 75.6%, respectively. Additionally, the multivariate analysis revealed that high entropy was an independent risk factor of ETE (odds ratio, OR = 19.348; 95%CI, 4.578-81.760; *P* = .001). The findings indicate a significant association between texture features of the primary tumor based on T2WI and the presence of ETE in PTC. These results have the potential to help predict ETE preoperatively in patients with PTC, offering valuable insights for clinical decision-making.

## 1. Introduction

The prevalence of papillary thyroid cancer (PTC), as a major subtype of well-differentiated thyroid cancer, has been steadily rising nowadays. Recent studies showed that PTC represents about 90% of all thyroid malignancy cases.^[1–3]^ Although PTC has a slow growth and low mortality, some aggressive behaviors such as extrathyroidal extension (ETE) are associated with poor outcomes, including local recurrence and distant metastases.^[4,5]^

Extrathyroidal extension, defined as tumor growth outside the thyroid gland and into surrounding tissues, occurs in up to 45% of PTC patients.^[1]^ Staging was by American Joint Committee on Cancer (AJCC) TNM classification for thyroid carcinomas, ETE is stratified into minimal ETE (invades to sternothyroid muscle or peri-thyroid soft tissues), and extensive ETE (invades to subcutaneous soft tissue, trachea, larynx, esophagus, recurrent laryngeal nerve, prevertebral fascia, carotid artery, or mediastinal vessels).^[6]^ In patients with PTC, minimal ETE is a strong predictor of lymph node metastasis and increased risk of postsurgical disease.^[7]^ The odds of recurrence in PTC with extensive ETE are 5 times higher than minimal ETE.^[7]^ Furthermore, a study with a larger sample size revealed that patients with minimal and extensive ETE were more likely to have larger tumors, lymphovascular invasion, and regional lymph node metastases than patients without ETE.^[8]^ Moreover, extensive ETE was associated with compromised survival for patients with PTC.^[8]^ The practice guidelines of the National Comprehensive Cancer Network (NCCN) for Thyroid Carcinoma recommend a total thyroidectomy for patients with extensive ETE.^[9]^ Therefore, it is ideal to have a noninvasive imaging method in identifying PTC with ETE, not only for accurate stage assessment preoperatively but also for optimal surgical strategy determination.

Ultrasound is the most commonly utilized imaging modality for preoperative PTC diagnosis and clinical staging. It is in essential a subjective assessment which relies on operator’s experience level.^[10,11]^ In comparison, CT is more objective in evaluating the invasion of PTC in adjacent tissues. However, previous studies failed to prove the diagnostic robustness of CT in predicting ETE relative to US.^[12,13]^ Magnetic resonance imaging (MRI) is a well-known noninvasive imaging technique for superior spatial resolution and excellent tissue contrast. Anatomic MRI has been mainly applied for preoperative PTC assessment.^[11,14]^ The corresponding ability is however limited in discriminating ETE.

As an alternative, texture analysis is a promising method for extracting quantitative features from medical images and further used these features to detect pathological changes that cannot be perceived by visional evaluation from radiologists.^[15]^ Previous studies showed high reliability of texture analysis in differentiating thyroid malignant nodules from benign as well as comparing negative and positive lymph nodal in PTC.^[16,17]^ Meyer et al showed that MRI texture features derived from T2-weighted imaging (T2WI) correlated with cell count and Ki67 in thyroid cancer.^[18]^ However, to our knowledge, no study has been performed to investigate if T2WI derived texture features are possible for predicting the extent of ETE in PTC.

Therefore, the main goal of this study was to investigate if T2WI derived histogram and gray-level co-occurrence matrix (GLCM) texture features are feasible in predicting the existence of ETE in PTC patients preoperatively.

## 2. Materials and methods

### 2.1. Study population

This retrospective study was approved by the local ethics committee of our university hospital, and the requirement for informed consent from recruited patients was waived.

A total of 104 patients with PTC confirmed by pathology in our hospital between January 2015 and April 2018 were enrolled in this study and underwent MRI examinations preoperatively. Each patient also underwent ipsilateral lobectomy(n = 63) or total thyroidectomy(n = 41). The time interval between MRI study and thyroidectomy was less than 2 weeks. Tissue samplings were collected from lesions during surgery and served as histopathological reference for ETE status assessment. Exclusion criteria of patients were defined in this study: No clearly visible images of tumor. The maximum diameter of primary tumor < 5 mm. Multiple lesions without one-to-one matching between the pathologic findings and tumors. Accompanied diseases such as nodular goiter, chronic lymphocytic thyroiditis or other thyroid diseases. Finally, a total of 76 PTC patients were selected for further data analysis. Moreover, according to the histopathological diagnosis, these patients were divided as without ETE (n = 45), with minimal ETE (n = 17), and with extensive ETE (n = 14).

### 2.2. MR image acquisition

All MRI experiments were performed on 1.5 Tesla MR scanner (GE Signa HD 1.5 T MR scanner; GE Healthcare Systems, Milwaukee, WI) with an eight-channel high-resolution receiver synergy-head/neck phased-array coil.

MRI protocols included transverse T1WI, transverse T2WI, diffusion weighted imaging and contrast-enhanced MRI. The spin echo-based T1WI sequence [repetition time (TR)/echo time (TE) = 520/14 ms, matrix size = 128 × 128, field of view (FOV) = 140 × 140 mm^2^; slice thickness = 3 mm; spacing between slices = 1 mm; scan time = 2 min 21s] in axial view and the fast spin echo-based T2WI sequence (TR/TE = 3500/95 ms, matrix size = 128 × 128, field of view (FOV) = 140 × 140 mm^2^; slice thickness = 3 mm; spacing between slices = 1 mm; scan time = 1 min 51 s and 2 min 40 s) in axial and coronal views were employed for imaging acquisitions. Axial DWI sequences comprising 4 b-values (0, 300, 500, and 800 s/mm^2^) were acquired, with the scanning plane centered around the thyroid gland with the number of excitations = 4 and the scan time of 5 min 59 s. Of all patients, contrast-enhanced T1WI (TR/TE = 520/14 ms, slice thickness = 3 mm, slice gap = 1 mm, field of view (FOV) = 40 cm^2^ × 28 cm^2^; matrix size = 256 × 256, number of excitations = 4, scan time = 5 min–6 min) was obtained with or without fat suppression immediately after the administration of 0.1 mmol/kg gadolinium-diethylenetriamine penta-acetic acid (Gd-DTPA) at an intravenous injection speed of 1.5 mL/s (Magnevist, Schering AG, Germany). Total scan time was approximately 30 minutes.

### 2.3. Image analysis

Two well-trained experienced (5–20 years) radiologists (H.S.D and Z.H) was employed to assess all acquired MR images and reached a consensus while being blinded to the pathological results. The largest diameter obtained from either sagittal or transverse views was recorded as the maximum tumor diameter. Based on the guidelines of the AJCC, minimal ETE was defined as the percentage of the primary tumor perimeter in contact with the thyroid capsule was > 25%; extensive ETE was defined as an extension of a tumor beyond the thyroid capsule and invading into the subcutaneous soft tissues, larynx, trachea, esophagus, recurrent laryngeal nerve, carotid artery, or mediastinal vessels.

**Figure 1.**
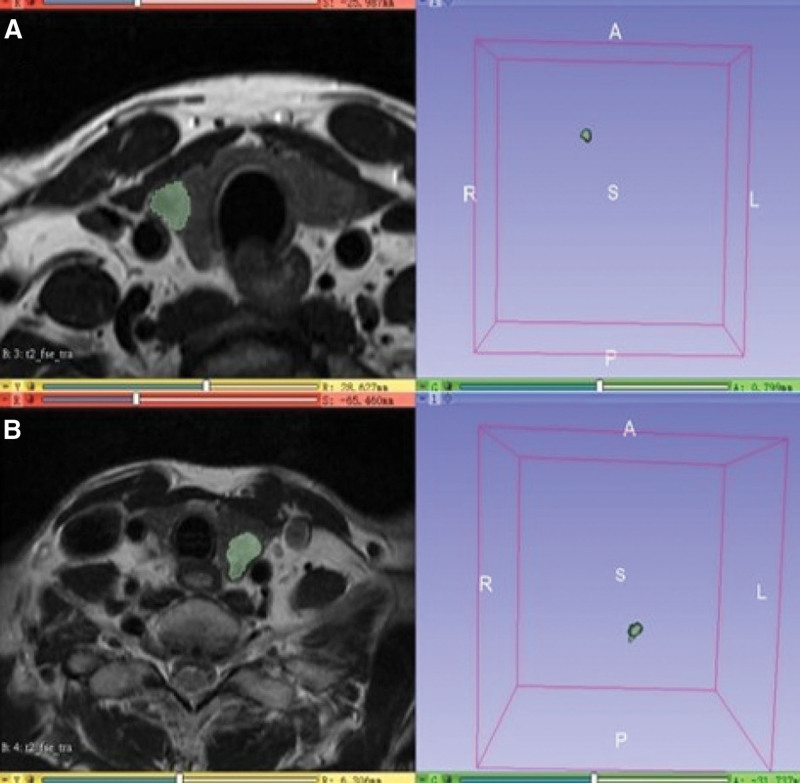
ROI segmentation based on transverse T2WI. (A) Image from a representative PTC patient without ETE (female, 47 years old). (B) Image from a representative patient with ETE (female, 37 years old). ETE = extrathyroidal extension, PTC = papillary thyroid carcinoma, ROC = receiver operating characteristic, T2WI = T2-weighted imaging.

Texture analysis was performed independently on axial T2WI using 3D slicer (Version V4.10.0) by 2 senior radiologists (Z.H and H.S.D, with 5 and 20 years of experience in head and neck MRI) while being blinded to the pathological results. Regions of interest (ROIs) covering the whole tumor were manually delineated along the tumor contour on each section, excluding obvious necrosis and cystic areas (Fig. 1). For each ROI, gray-level normalization was performed, using the limitation of dynamics to μ ± 3 SD (μ gray level mean, SD standard deviation) to minimize the influence of contrast and brightness variation. Nine histogram and GLCM texture features were automatically extracted, including mean value, standard deviation, skewness, kurtosis, entropy, energy, correlation, contrast, and inverse difference moment.

### 2.4. Histopathological analysis

Two pathologists reviewed the hematoxylin and eosin section of each PTC and evaluated tumor aggressiveness according to the American Thyroid Association guidelines. Based on histopathological examinations, all PTC patients were divided into 3 groups: without ETE, minimal ETE (tumor invasion beyond the thyroid capsule identified at pathological examination), and extensive ETE (gross tumor invasion identified at surgery and confirmed by histopathological review).

### 2.5. Statistical analysis

All statistical analyses were performed in SPSS 17.0 software (version 17; IBM, Armonk, NY). Categorical variables were compared among 3 different PTC patient groups using the chi-square test. Comparisons of age, maximum diameter of tumor and texture parameters among different patient groups were performed using the independent-samples *t* test or Mann–Whitney *U* test depending on distribution normality or non-normality, respectively. Spearman correlation analysis was separately applied to assess the correlation between each texture parameter and ETE. Subsequently, multivariate binary logistic regression analysis was performed to identify independent predictors for ETE. Interobserver agreement of assessing Z.H. between H.S.D radiologists was evaluated using the intraclass correlation coefficient (ICC). An ICC value above 0.75 was considered excellent agreement. Receiver operating characteristic (ROC) curve analysis was performed to assess the diagnostic performance of each texture parameter in predicting ETE status by calculating the area under the ROC curve (AUC). *P* < .05 was considered statistical significance.

## 3. Results

### 3.1. Patient characteristics

Clinicopathological features of total 76 PTC patients (mean age 46.11 ± 11.68 years, 63.2% female) with and without ETE were included in our study (Table 1). According to the operative and pathologic findings, 45 patients were diagnosed without ETE, and the rest 31 patients were diagnosed with ETE. Furthermore, in 31 patients with ETE, 17 patients were determined with minimal ETE and 14 patients with extensive ETE. No significant statistical difference was observed for any subgroups for the clinicopathological features except primary tumor size (Tables 1 and 2).

**Table 1:** Clinicopathological features of 76 PTC patients (97 nodules) with and without ETE.

	ETE	Without ETE	*P* value
Age (yr)	43.27 ± 11.89	48.62 ± 12.07	.144
Gender			
Female	20	28	.301
Male	11	17	
Primary tumor size(cm)	1.65 ± 0.68	1.14 ± 0.45	.001
Lymph node metastasis	17	30	.634
Tumor location			
Right thyroid	21	32	.713
Left thyroid	17	23	
Isthmus	2	2	

ETE = extrathyroidal extension, PTC = papillary thyroid carcinoma.

**Table 2:** Clinicopathological features of 31 PTC patients (40 nodules) with minimal ETE and extensive ETE.

	Minimal ETE	Extensive ETE	*P* value
Age(years)	47.91 ± 11.43	45.34 ± 12.94	.517
Gender			.301
Female	13	12	.247
Male	4	2	
Primary tumor size(cm)	1.35 ± 0.68	1.77 ± 0.34	.037
Lymph node metastasis	11	14	.649
Tumor location			
Right thyroid	11	5	.161
Left thyroid	11	9	
Isthmus	3	1	

ETE = extrathyroidal extension, PTC = papillary thyroid carcinoma.

### 3.2. Interobserver agreement

Interobserver agreement between both radiologists was excellent for assessing all texture features derived from separately delineated ROIs. The obtained ICC values ranged from 0.78 to 0.89.

### 3.3. Texture parameter analysis

The histogram and GLCM parameters between 2 groups were compared and shown in Table 3. Significantly lower energy (0.27 vs 0.51, *P* < .001) and correlation (0.31 vs 0.63, *P* = .001) as well as higher entropy (6.40 ± 0.71 vs 5.60 ± 0.40, *P* < .001) and standard deviation (11.47 vs 8.19, *P* = .001) were found in PTC patients with ETE than without ETE group (Table 3). No significant differences of the texture parameters were found between minimal ETE and extensive ETE groups.

**Table 3:** Histogram and GLCM parameters between PTC patient groups with and without ETE.

Variable	ETE	Without ETE	*P* value
Entropy	6.40 ± 0.71	5.60 ± 0.40	.000
Standard deviation	11.47 (8.23, 15.67)	8.19 (5.49, 11.45)	.001
Energy (×10^−2^)	0.27 (0.15, 0.38)	0.51 (0.33, 0.71)	.000
Correlation (×10^−2^)	0.31 (0.25, 0.75)	0.63 (0.33, 1.15)	.001
Mean value	65.50 ± 24.94	58.12 ± 20.97	.059
Skewness	0.09 ± 0.57	0.17 ± 0.57	.442
Kurtosis	0.18 ± 0.54	0.32 ± 0.49	.269
Contrast	82.51 ± 79.57	69.11 ± 121.38	.471
Inverse difference moment	0.21 ± 0.08	0.23 ± 0.07	.129

ETE = extrathyroidal extension, GLCM = gray-level co-occurrence matrix, PTC = papillary thyroid carcinoma.

**Figure 2.**
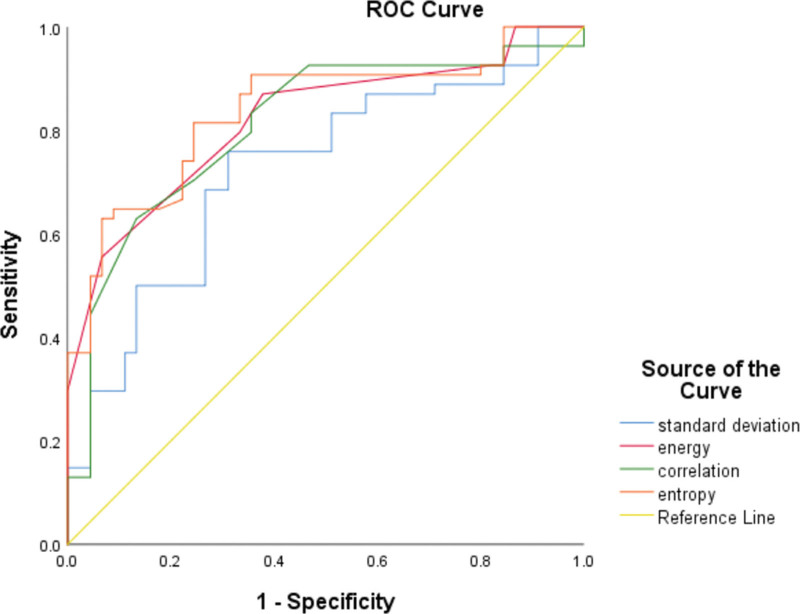
ROC curves of texture parameters in predicting ETE of PTC. ETE = extrathyroidal extension, PTC = papillary thyroid carcinoma, ROC = receiver operating characteristic.

The primary tumor size, energy, entropy, correlation and standard deviation were preliminarily selected as covariates for multivariate logistic regression analysis. High entropy was thus considered an independent risk factor of ETE (odds ratio, OR = 19.348; 95% CI, 4.578–81.760; *P* = .001; Table 4). Using ROC analysis, entropy showed moderate to good diagnostic power with a cutoff value > 5.86 (AUC = 0.837, 95% CI, 0.764–0.910) in predicting ETE, yielding sensitivities of 81.5% and specificities of 75.6% (Fig. 2).

**Table 4:** Multivariate logistic regression analysis results.

Variable	OR	95% confidence interval (CI)	*P* value
Primary tumor size (cm)	4.972	3.231–11.479	.081
Entropy	19.348	4.578–81.760	.001
Standard deviation	1.113	0.954–1.298	.175
Energy	1.880	0.038–92.411	.751
Correlation	0.853	0.180–4.042	.841

CI = confidence interval, OR = odds ratio.

## 4. Discussion

Currently, preoperative risk prediction of ETE in PTC patients remains challenging. The incidence of ETE is reported between 5% to 45%,^[7,19]^ whereas plays one of the most important reasons for increased risk for morbidity and mortality. Noninvasive, accurate and low-cost assessment of ETE in PTC before surgery has the potential clinical implications. In this study, we acquired transverse T2WI images of PTC patients preoperatively and performed texture analysis on whole tumor. The extract texture features were further investigated for the relationship with the ETE status of PTC patients. As shown in the results, the texture feature of entropy, energy, correlation and standard deviation derived from T2WI showed significant correlated with ETE, but only entropy was the independent risk factor for predicting ETE status for PTC patients.

In texture analysis, texture features were extracted from medical image based on whole-lesion ROI, which is used to quantitatively characterize the intra-tumoral heterogeneity by calculating the distribution.^[20]^ Some studies have indicated that texture analysis based on T2WI image has potential tumor diagnostic and prognostic evaluation values.^[21–23]^ In comparison to other MRI sequence, T2WI has long TE and increase the imaging contrast between tissues. This may be the reason why most studies choose T2WI images for texture analysis. However, the correlation between texture features derived from T2WI and ETE of PTC remains unclear.

Our study found that patients without ETE had significantly higher energy and correlation, and lower standard deviation and entropy in tumor than with ETE. Interobserver variability of texture analysis between 2 radiologists was also assessed in our study. An overall excellent interobserver agreement was found, with ICCs ranging from 0.78 to 0.89. This proves that statistical-based histogram and GLCM texture parameters had good repeatability in clinical practice. Correlation indicates the directionality of the texture. Standard deviation describes the variation from the mean pixel value. Yun et al^[24]^ reported lower correlation and higher standard deviation in pancreas head cancer patients with than without recurrence. This is consistent with the results of our current study. Both Yun et al^[24]^ and us have demonstrated that lower correlation and higher standard deviation is found in more invasive tumors. High energy reflects that the distribution of intensity levels is narrow. Entropy is the opposite index to energy and reflects the complexity of lesion texture. Yang et al^[25]^ studied T2WI derived textural characteristics of eighty-eight rectal adenocarcinoma patients and found that no lymph node metastasis group had a significantly higher energy and a lower entropy than lymph node metastasis group. We found that standard deviation and entropy level represented the complexity of the texture in the lesion ROI, which showed larger value with increased tumor heterogeneity. Heterogeneity of the tumor microenvironment is a well-recognized feature of malignancy that is associated with adverse tumor biology, reflecting changes in cell permeability, abnormal angiogenesis, and changes in tissue structure caused by mucus-like changes, necrosis, and fibrosis.^[26]^ These subtle pathological changes are invisible to mortal eyes, but can revealed in texture analysis features. Multivariate analysis showed that entropy was an independent risk factor of ETE with an OR of 19.348. Entropy represents the randomness or heterogeneity of the pixel distribution, and thus high entropy distinguishes hidden microcosmic heterogeneity between PTC patients with and without ETE. Recent work on mass-like breast cancer also reported the value of entropy in evaluating pathologic complete response and predicting survival outcomes.^[27]^ No significant difference was however found in all texture features of histogram and GLCM between minimal ETE and extensive ETE. This may be probably due to not large sample size applied in this study. We expect that our method will be helpful for preoperative prediction ETE of PTC. Therefore, a large clinical cohort is requested to further validate in a large sample and a multicenter study in the future.

Conventional imaging techniques have been so far widely applied to assess the morphology of PTC preoperatively.^[11,13,28]^ Gweon et al^[10]^ found that the overall accuracy rates (60.8% and 66.2%) as well as diagnostic specificity are low in predicting ETE on 2D or 3D sonography. Moreover, there is still a lack of unified diagnostic criteria for ultrasonography for assessing extension. Kwak et al^[29]^ suggests that more than 25% of tumor perimeter contact with the adjacent capsule was the most accurate measurement for predicting ETE, but Park et al^[30]^considered that more than 50% tumor abutment with the thyroid capsule was optimal. Iodinated contrast media is a source of excess iodine that may induce thyroid dysfunction, so that, CT was not suggested to evaluate PTC mainly. Our previous work demonstrated that MRI is a noninvasive method in diagnostic ETE which exhibited good specificity but a low sensitivity. The selection bias from the subjective judgment of physicians should not be ignored. Preoperative clinical diagnosis is difficult due to low sensitivity reported by previous imaging studies. Compared with conventional imaging techniques, texture analysis has significantly improved the sensitivity of diagnosis ETE in PTC. Moreover, one advantage of our study is that the texture features of whole PTC lesion rather than of the lesion at the maximum layer has been outlined.

This study has some limitations. Firstly, the sample size is limited and from a single imaging center, large clinical cohorts better from multi-center should be investigated in future studies. Secondly, this study has a retrospective nature. Thirdly, only anatomic T2WI images were included for texture analysis, although this technique has been the most widely applied for tumor diagnosis in clinic. Other MR functional imaging techniques, such as diffusion weighted imaging, should be included to provide more comprehensive information of tumors in future studies.

In summary, texture features extracted from T2WI might be considered potential valuable imaging biomarkers in predicting the ETE state of PTC. The ability to predict the presence of ETE using texture analysis will allow the clinician to identify patients that are likely to benefit from more aggressive initial therapy, particularly in low-risk PTC.

## Author contributions

**Conceptualization:** Chengjia Qian, Shan Chen, Shudong Hu.

**Data curation:** Chengjia Qian, Shan Chen, Li Liu.

**Formal analysis:** Chengjia Qian, Shan Chen, Li Liu, Heng Zhang.

**Funding acquisition:** Chengjia Qian, Heng Zhang.

**Investigation:** Chengjia Qian, Shan Chen, Heng Zhang.

**Methodology:** Chengjia Qian, Shan Chen, Heng Zhang.

**Project administration:** Chengjia Qian, Shan Chen, Li Liu, Shudong Hu.

**Resources:** Chengjia Qian, Li Liu, Weiqiang Dou.

**Software:** Weiqiang Dou.

**Supervision:** Heng Zhang.

**Visualization:** Shudong Hu.

**Writing – original draft:** Chengjia Qian, Shan Chen.

**Writing – review & editing:** Chengjia Qian, Shudong Hu, Heng Zhang.
